# Comparing intraoperative ultrasound and wire-guided localisation for skin cancer metastases: impacts on patient safety, outcomes, costs, and implementation

**DOI:** 10.1186/s12957-026-04389-1

**Published:** 2026-05-21

**Authors:** Nadin Hawwash, Gregory Royle, Damian Mullan, Damir Kosutic, Kantappa Gajanan

**Affiliations:** 1 School of Medicine, Faculty of Biology, Medicine and Health, University of Manchester, Manchester, UK; 2 Departments of Radiology, The Christie NHS Foundation Trust, Manchester, UK; 3 Departments of Plastic Surgery, The Christie NHS Foundation Trust, Manchester, UK

## Abstract

**Aims:**

Localisation of non-palpable isolated or oligometastatic disease can be an intraoperative challenge. This quality improvement study aims to demonstrate the novel use of intraoperative ultrasound (IOUS) in localising oligometastatic disease. We evaluated its clinical effectiveness in comparison with the gold-standard wire-guided localisation technique in localising oligometastatic disease.

**Methods:**

We assembled a database of patients at a specialist tertiary referral centre in the United Kingdom who had IOUS-guided or wire-guided tumour localisation and conducted informal interviews with two Plastic Surgery Consultants, a Consultant Radiologist and a Radiographer. We retrospectively analysed the effectiveness of IOUS guidance in localising cancer metastasis, focusing on physician-reported patient safety, clinical outcomes, costs and implementation and compared this to the wire-guided technique.

**Results:**

Over the last 8 years, 19 patients had IOUS-guided tumour localisation, and 8 patients had wire-guided localisation since its on-site implementation 3 years ago. A retrospective cohort analysis was performed comparing 8 patients who underwent wire-guided tumour localisation between 2022 and 2024 with 8 patients who received IOUS-guided tumour localisation over the same period. Although IOUS extended the duration of surgery, it was less invasive, associated with lower complication rates (12.5% vs. 25.0%) and offered real-time reassurance. IOUS use was £12.65 (18%) cheaper and easier to implement compared with wire-guided localisation. Since 2017, IOUS-guided localisation has been associated with a low positive margin rate of 5.26% and a re-excision rate of 11.1%.

**Conclusion:**

The advantages of using IOUS for patient safety, cost-effectiveness and overall outcomes highlight its role in clinical practice and the need for a standardised protocol for its implementation. Conducting a large-scale multi-centre study is necessary to improve the generalisability of findings.

## Introduction

Surgical excision of isolated or oligometastatic disease remains the gold standard for management [1]. The primary goal is to achieve complete tumour resection, ensuring negative margins while sparing normal tissue. Prior localisation of disease is necessary and achieved by palpation or visual localisation. Static pre-operative imaging used to assess metastasis and guide management includes Computed Tomography (CT), whole-body Positron Emission Tomography-Computed Tomography (PET-CT), ultrasonography (USS) and magnetic resonance imaging (MRI) [2]. From 1998 to 2012, a study on the National Cancer Database found that such localisation methods achieved a 12.75% pooled positive margin rate [3]. It is crucial to minimise the incidence of positive margins by ensuring complete resection with adequate margins of healthy tissue due to implications for recurrence and reduced overall survival [4]. However, impalpable or ill-defined lesions, especially those at anatomically challenging sites, pose an intraoperative challenge in ensuring complete excision. It requires real-time guidance to confirm lesion boundaries and margins and provide intraoperative reassurance of complete resection with no residual disease. Techniques to guide tumour excision include wire-guided, intra-operative ultrasound, magnetic, fluorescence, and radio-occult lesion localisation [5–8]. Although various techniques have been established, this paper compares intraoperative ultrasound (IOUS) use with current practice using wire-guided localisation (WGL), given the promising real-time reassurance associated with IOUS-guided localisation of oligometastatic disease.

### Wire-guided localisation

WGL methods have been used since the 1970s to localise oligometastatic disease by pre-operative marking the lesion with wires protruding from the skin under USS, mammographic or MRI guidance by a radiologist in the ultrasound suite [9]. Collaborative planning between the surgeon and radiologist is necessary to ensure localisation on the day of surgery. First, pre-operative imaging is used to guide tumour marking. Local anaesthetic is administered at the site of the lesion, a transducer is positioned above the lesion and a guide-wire needle is inserted 4 cm laterally away from the transducer [10]. The guidewire hooks are passed through the mass lesion. The lesion borders are marked, and the external hook wire is fixed onto the skin [10]. Although the WGL is beneficial in its common use and precise target for surgical excision of non-palpable tumours, its drawbacks include patient discomfort, infection and injury to the site of wire insertion, potential wire displacement and migration, and the risk of a needle stick injury posed to radiologists and surgeons involved, especially when working with high-risk patients such as those with HIV [11]. Residual disease following lesion removal using WGL cannot be excluded in real time, posing the potential need for subsequent surgery for residual disease, likely to increase patient morbidity.

### Intra-operative ultrasound localisation

IOUS can facilitate lesion detection and complete metastasectomy by providing real-time information [12]. An experienced radiologist with the necessary equipment and scanning technique is required to achieve the necessary image quality and interpretation, which cannot be achieved by a surgeon alone, particularly for anatomically challenging locations. The radiologist must be scrubbed, gowned and gloved in theatre to maintain a sterile field. Prior review of pre-operative imaging by the radiographer is necessary to note lesion location concerning identifiable landmarks and decide on the appropriate transducer to use. Intraoperative probes are typically smaller to access anatomical regions with limited accessibility and require sterile preparation, typically using sterile condom sheaths [12]. A transducer is positioned directly over the suspected lesion site to localise the lesion. Scanning typically takes between 5 and 10 min, depending on the radiologist’s skill set and may require light dimming to aid visualisation. Tumour localisation and margins are discussed with the surgeon and marked. Following tumour excision, rescanning ensures complete resection given the absence of tissue heterogeneity in the tumour bed, which would indicate residual disease.

The literature demonstrating the efficacy of IOUS use for metastasectomy is limited. Only eight out of thirty-five article results were relevant following a PubMed search using the terms “intraoperative ultrasound” AND (“melanoma” OR “squamous cell carcinoma” OR “SCC” OR “sarcoma” OR “basal cell carcinoma” OR “BCC” OR “metastasectomy”) (Tables 1 and 2). All eight studies demonstrated the beneficial use of IOUS for oropharyngeal squamous cell carcinoma [13], axillary and supraclavicular lymphadenectomy [14], gynaecologic cancer patients with isolated recurrent disease [15], and cerebral metastasis [16]. Two studies showed the beneficial use of IOUS in soft tissue tumours [17, 18] and two studies demonstrated use for oral tongue tumours [19, 20]. Although the deep margin clearance was non-significantly improved in the IOUS group in the study by Bulbul et al., for tongue carcinoma resection [20], the lack of statistical significance could be due to the small sample size limiting statistical power to detect modest differences. Generally, it is important to acknowledge metastasectomy cases suitable for excision are limited to a specific group of patients; therefore, sample sizes for studies on IOUS use will be limited especially in single-site studies.

A network meta-analysis of randomised controlled trials based in the Netherlands, Germany and China by Davey et al., comparing WGL versus IOUS-guided localisation found IOUS use to have achieved significantly lower margin positivity (odds ratio (OR): 0.192, 95% confidence interval (CI): 0.079–0.450) and reoperation rates (OR: 0.182, 95% CI: 0.069–0.434) compared to WGL [21]. However, to our knowledge, no study has compared IOUS with WGL in the United Kingdom (UK). Although current evidence outlines the benefit of using IOUS in practice and Lubner et al., outline techniques to optimise intraoperative USS use, there is no standardised protocol or standard operating procedure (SOP) for its use in the UK [12].

### Aims

This quality improvement study aims to compare traditional WGL with IOUS localisation on a patient safety, clinical outcomes, costs and implementation level.

**Table 1:** Relevant studies in the literature on the intraoperative ultrasound guidance technique

Study, date	Country	Method	Relevant summary taken directly from abstracts
Kishikawa et al., (2025) [13]	Japan	Case report on IOUS use in photoimmunotherapy in a 70 year old male with oropharyngeal squamous cell carcinoma with superficial cervical lymph node recurrence.	“IOUS confirmed the localization of the lesion and major vessels near the tumour.”
Gajanan et al., (2023) [22]	United Kingdom	Case report of 35-year-old male patient demonstrating use of IOUS for axillary and supraclavicular lymphadenectomy for advanced metastatic melanoma.	“IOUS can help guide surgeons trying to achieve clearance of metastatic disease in anatomically complex regions, avoiding unnecessary morbidity.”
Bulbul et al., (2022) [20]	United states	Retrospectively compared use of IOUS guided excision of oral tongue carcinoma in 23 patients to 21 patients without intraoperative imaging guidance.	“The mean deep margin clearance was 8.5 ± 4.9 and 6.7 ± 3.8 for the IOUS and non- IOUS groups respectively (p-value 0.18) showing a non-significant improvement in the IOUS group
Yoon et al., (2021) [23]	United States	Retrospective analysis of the accuracy of pre-operative CT for oral tongue squamous cell carcinoma tumour thickness measured in 19 patients was compared to IOUS determined tumour thickness.	“Preoperative CT is not reliable for assessment of tumour thickness in oral tongue squamous cell carcinoma compared to IOUS and histopathology, particularly for oral tongue squamous cell under 10 mm.” (23)
Takeuchi et al., (2020) [18]	Japan	Retrospective analysis of 19 patients who had IOUS localisation of soft tissue sarcoma that was impalpable, ill- defined, or beneath the fascia	“The recurrence-free survival rate was 88.9% in 5 years.” “IOUS-guided surgery is useful for visualizing soft tissue sarcoma.”
Mascilini et al., (2018) [15]	Canada	Prospective study of 51 gynaecologic cancer patients with isolated recurrent disease. 12/51 women needed IOUS to identify the lesions.	“About 1 of 4 patients (25%) with single gynaecologic cancer recurrence needs IOUS to benefit from minimally invasive surgery for complete secondary cytoreduction.”
Oliveira et al., (2017) [16]	Brazil	Compared Karnofsky index scores and tumour volume of cerebral metastasis of 35 patients with and 43 without IOUS.	“The residual tumour volume was lower in the IOUS group (9.5% and 1.6 mm3 vs. 30.8% and 9 mm3, respectively; P[ 0.05).”
Farfalli et al., (2011) [17]	Argentina	Retrospectively analysed efficacy of IOUS in excising 22 impalpable musculoskeletal soft tissue tumours.	“The procedure allowed recognized and excised additional nodules not previously diagnosed in 3 cases.”

Abbreviations: *IOUS* intraoperative ultrasound, *CT* computed tomography

## Methods

### Interventions

We investigated the use of IOUS to localise the oligometastatic disease and compared this with traditional WGL.

### Study of interventions

The procedure and implementation of IOUS and WGL was discussed during an informal interview separately conducted with two consultant plastic surgeons, a radiologist and a radiographer. Data collected on patients who underwent IOUS localisation was compared with patients who underwent traditional WGL. Quality improvement through the use of IOUS localisation was evaluated based on four key themes:

1. Physician-reported patient safety
2. Clinical outcomes
3. Costs
4. Implementation

Clinical outcomes such as margin positivity rates, reoperation rates and postoperative complications were compared. To assess the sustainability of IOUS localisation in practice, costs for equipment and running were analysed. Factors involved in implementation (surgical time, training, standardised protocol and documentation) were compared.

### Interviews

Interviews were conducted by the first author (a clinician-researcher) and consisted of non-structured discussions with key stakeholders, including two Consultant Plastic Surgeons, one Consultant Radiologist, and one Radiographer, within a clinical setting to capture expert perspectives relevant to the study. The interviews were conducted in a non-structured format but guided by predefined thematic domains, allowing participants to discuss their overall clinical experience with each of the two techniques (IOUS and WGL). Topics explored included physician-reported patient safety, localisation accuracy, complication rates, and implementation factors such as localisation time, equipment requirements, and procedure duration. Key insights from the informal expert discussions were summarised to provide context regarding clinical experience with each of the two techniques.

### Measures

Data on patients who underwent IOUS or WGL on-site was retrospectively collected from the Clinical Web Portal (CWP). Since there was no code for IOUS, cases were identified by matching USS dates with the surgery date and two consultant surgeons’ names to create a list of patients with a same-day USS. Operative notes were then reviewed to identify IOUS cases. Similarly, because no code existed to identify patients who had WGL, manual identification through reviewing operative notes was necessary. Variables collected included age, primary disease site, cancer stage and type, date of surgery, histology, margin positivity, cancer recurrence, treatment status for cancer, and current disease status.

This study is reported using Standards for Quality Improvement Reporting Excellence guidelines [24].

## Results

Nineteen patients had IOUS localisation between 2017 and 2025 (Tables 1 and 2) of which over 2022–2024, 8 had IOUS localisation (Table 3a) and 8 had WGL (Table 3b). Focusing on patients over 2022–2024, the mean age of IOUS and wire-guided patients were 62.1 years (standard deviation (SD) 15.0) and 55.6 years (SD 16.9), respectively. A summary description of patients included for IOUS localisation and WGL is provided in Table 3. The metastasectomy regions marked using IOUS guidance included 3 in the head and neck, 4 in the upper limb, 7 in the lower limb, and 4 in the trunk (Tables 1 and 2). No WGL cases included the head and neck region, 1 included the upper limb region, 2 included the lower limb, and 5 included the trunk region (Table 3). IOUS was used to guide the excision of melanoma primarily with only one squamous cell carcinoma (SCC) case and one sarcoma case (Tables 1 and 2). WGL was used to guide the excision of 5 melanoma cases and a case of mycosis fungoides, leiomyosarcoma and metastatic ependymoma (Table 3).

**Fig. 1:**
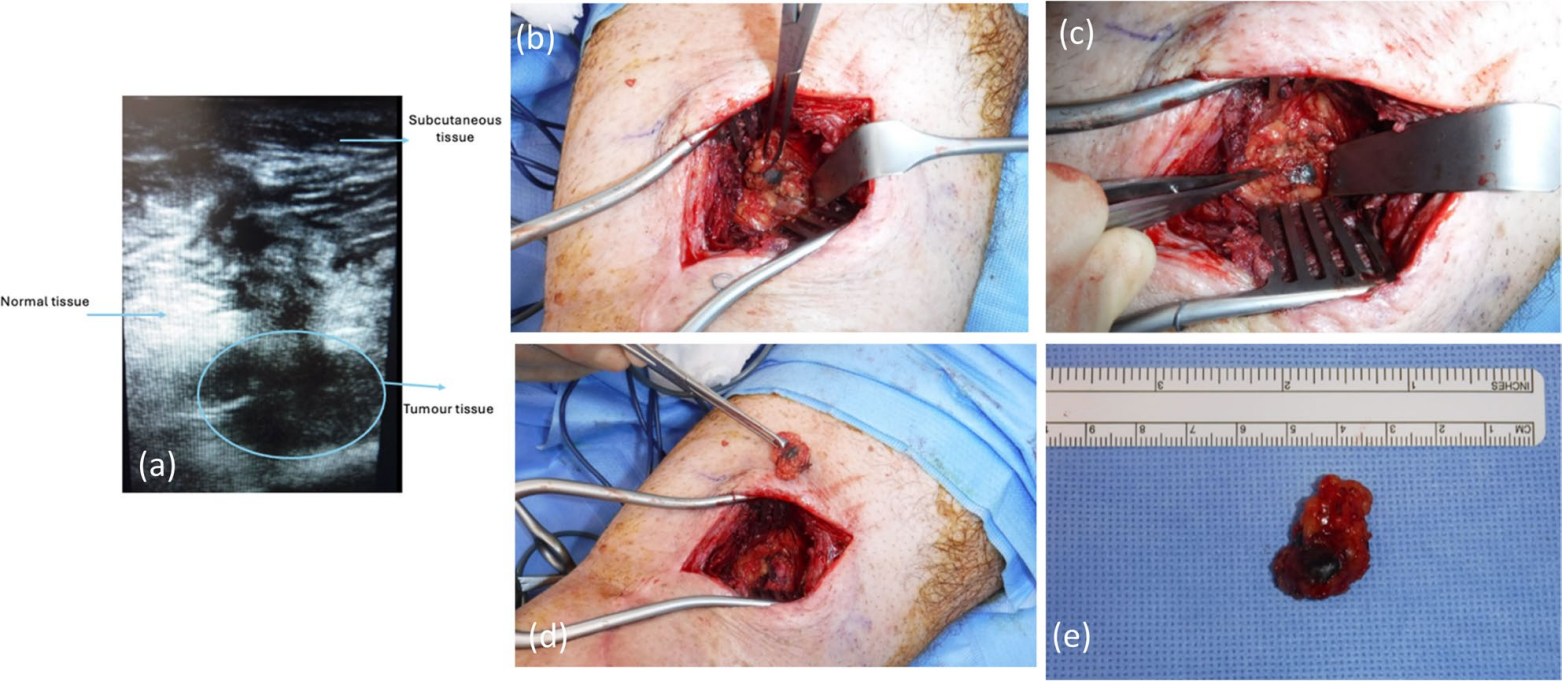
An example demonstrating the use of intraoperative ultrasound to guide tumour excision; (**a**) tumour identified on PET CT scan was intraoperatively localised with a portable ultrasound scan, (**b**) an initial excision at lesion site was made and the neurovascular bundle was retracted, (**c**) intraoperative ultrasound visualised tumour site and guided excision, (**d**) tumour excised (**e**) tumour measure around 1 × 1 cm

An example of the use of intraoperative ultrasound is provided in Fig. 1.

**Table 2:** Summary of all patient cases that had intraoperative ultrasound localisation

Patient Number	Age	Cancer type	Cancer stage	Lesion location	Margin status	Re-excision required	Complication
1	56	Melanoma	NA	Right heel	Negative	No	None
2	64	Melanoma	3c	Right upper back	Negative	No	None
3	61	Melanoma	4	Left scapula	Negative	No	None
4	78	Melanoma	3c	Right calf	Negative	No	None
5	79	SCC	4	Right parotid	Negative	No	Seroma in ear
6	51	Myxo-inflammatory sarcoma	4	Left lower leg	Negative	No	None
7	57	Melanoma	3c	Left shoulder	Negative	No	None
8	35	Melanoma	2a	Left flank	Negative	Yes	None
9	59	BCC	NA	Posterior Neck	Negative	No	None
10	64	Melanoma	3b	Right axilla	Negative	No	None
11	74	Melanoma	NA	Left upper abdominal wall, right inguinal area	Negative	No	None
12	53	Melanoma	NA	Left scapula	Negative	No	None
13	79	Melanoma	NA	Medial left thigh	Positive	No	None
14	48	Melanoma	4	Right breast	Negative	No	None
15	55	Melanoma	4	Left neck	Negative	Yes	None
16	44	Melanoma	4	Left leg	Negative	No	None
17	63	Melanoma	1b	Left leg	Negative	No	None
18	67	Melanoma	4	Right leg, right groin	Negative	Yes – different site (scalp)	Moderate lymphoedema right leg
19	35	Melanoma	3	Neck	Negative	Yes – different site	Chest wall oedema

Abbreviation: *SCC* squamous cell carcinoma, *NA* not available

**Table 3:** Summary of patient cases that had (a) intraoperative localisation and (b) wire-guided localisation 2022–2024

Patient Number	Age	Cancer type	Cancer stage	Lesion location	Margin status	Re-excision required	Complication
(a) Intraoperative ultrasound localisation							
1	78	Melanoma	3c	Right calf	Negative	No	None
2	79	SCC	4	Right parotid	Negative	No	Seroma in ear
3	51	Myxo-inflammatory sarcoma	4	Left lower leg	Negative	No	None
4	57	Melanoma	3c	Left shoulder	Negative	No	None
5	35	Melanoma	2a	Left flank	Negative	Yes	None
6	59	BCC	NA	Posterior Neck	Negative	No	None
7	64	Melanoma	3b	Right axilla	Negative	No	None
8	74	Melanoma	NA	Left upper abdominal wall, right inguinal area	Negative	No	None
(b) Wire-guided localisation							
1	74	Melanoma	3c	Right buttock	Negative	No	Seroma in right buttock
2	33	Melanoma	3b	Left chest	Negative	No	None
3	52	Mycosis Fungoides	NA	Open biopsy of right groin lymph node	Negative	No	Infection in right groin site
4	71	Leiomyosarcoma	NA	Left paraspinal muscles	NA	NA	None
5	74	Melanoma	4	Left subcostal area, right inguinal region	Negative	No	None
6	49	Melanoma	3b	Right axilla	Negative	No	None
7	59	Melanoma	3c	Right rectus sheath	Negative	No	None
8	33	Metastatic ependymoma	NA	Right flank	Negative	No	None

Abbreviation: *SCC* squamous cell carcinoma

### Patient safety

#### Physician-observed patient comfort

During informal interviews, one surgeon reported observing higher levels of anxiety experienced by patients who underwent WGL. Although local anaesthesia is administered beforehand, patients were perceived to experience pain and discomfort from the procedure. In contrast, IOUS localisation was described by the surgeons as non-invasive, pain-free, with patients under general anaesthesia so not experiencing any procedure-related anxiety or discomfort. These observations reflect physician-reported impressions rather than patient-reported outcome measures.

### Clinical outcome

#### Localisation precision

Both consultant surgeons who used IOUS localisation reported greater confidence due to real-time intraoperative guidance, which helped ensure complete lesion resection and achieve optimal surgical and deep resection margins, particularly in anatomically challenging sites. Immediate feedback on the absence of residual disease improved surgeon confidence, unlike WGL, which relied on histology and reimaging to determine if additional resection is necessary. IOUS provided real-time imaging of high-risk structures such as blood vessels, vascular structures and nerves, minimising accidental damage. One consultant surgeon noted the limited applicability of WGL in certain anatomical sites, as in one case, WGL could not be used to localise a lesion in the popliteal fossa given the proximity to important structures and potential wire-associated damage.

#### Margin status

Post-operative histology found that all patients who underwent IOUS localisation and WGL between 2022 and 2024 had a negative margin status. Since implementation in 2017, 1 out of 19 patients (5.3%) had a positive margin status post-op IOUS-guided localisation.

#### Reoperation rates

1 out of 8 patients (12.5%) who had IOUS localisation required same-site re-excision of a melanoma in the left flank, whereas no WGL cases over the 3 years required re-excision (Table 3). Since 2017, 2 out of 19 patients (10.5%) required a same-site re-excision following initial IOUS-guided localisation.

#### Complication rates

A surgeon noted that WGL is an invasive procedure that proves painful and poses a risk of bleeding and infection, less suitable in high-risk patients (patients with HIV or patients on blood thinners). Additionally, a surgeon noted complications may arise with wire migration and damage, whereas IOUS is a non-invasive technique that would be a more suitable localisation method, especially in high-risk patients. Over 2022–2024, only 1 out of 8 patients (12.5%) experienced a post-operative complication with IOUS localisation and 2 out of 8 WGL cases (25%) experienced complications, including seroma and infection (Table 3). Since implementation in 2017, 3 out of 18 IOUS-guided patients (16.7%) experienced complications, including seroma, lymphoedema and oedema, but these were eventually resolved (Tables 1 and 2).

### Costs

#### Equipment

High-quality ultrasound machines are required for WGL and IOUS use. At the specialist tertiary referral centre, a Samsung prestige RS85 machine LA2-9 S is used for WGL and a portable Venue Go 12 L-RS machine is used for IOUS localisation [25, 26]. The ultrasound unit cost of the Samsung Prestige is 62.2% higher than the Venue Go, costing around £34,587.60 and £21,320, respectively [25, 27] (Table 4).

#### Running costs

IOUS localisation outperforms the WGL technique, proving to be £12.65 cheaper per patient case (Table 4). Investing in IOUS training and upskilling surgeons to perform intraoperative localisation would further reduce costs by £25 per patient after the initial training investment is covered. IOUS localisation provides real-time feedback on whether complete resection is achieved, reducing the likelihood of subsequent surgery (and its associated costs) compared with the pre-operative WGL, which does include an opportunity to check for residual disease intraoperatively to ensure further excision of residual disease in the same surgery.

**Table 4:** Costings of (a) wire-guided ultrasound localisation versus (b) intraoperative ultrasound localisation

Open (small trolley)	N^o^	Value (pre VAT)	Total
(a)			
Biopsy pack	1	£4.48	£4.48
Small drape	1	£1.63	£1.63
10.5ml chloraprep (with tint)	1	£3.65	£3.65
USS probe cover	1	£3.02	£3.02
Lidocaine	1	£0.61	£0.61
10mls syringe	1	£0.18	£0.18
Kopans needle	1	£38	£38
Radiographic aide (band 3)	2	TBC (£12 per hour each)	£6
Consultant Radiologist	1	TBC (£51 per hour)	£12.75
Room time	-	15 minutes activity	NA
Total running cost	-	-	**£70.32**
Total equipment cost	**1**	**£34,587.60**	**£34,587.60**
(b)			
10.5ml chloraprep (with tint)	1	£3.65	£0*
USS probe cover	1	£3.02	£3.02
Consultant Radiologist	1	TBC (£50 per hour)	£25
Room time	-	60 minutes activity	NA
Total running cost	-	-	**£28.02**
Total equipment cost	**1**	**£21,320**	**£21,320**

* No associated chloraprep cost given already used for operation

Abbreviations: *USS* ultrasound, *NA *not applicable

#### Reoperation costs

Costs for reoperation associated with incomplete lesion excision with either method would be similar and thus not compared in depth.

### Implementation

#### Stakeholders

Stakeholders identified through discussion that would be affected by changes to lesion localisation methods include surgeons, radiologists, anaesthetists (particularly regarding positioning requirements and changes in procedure duration), theatre staff, oncologists, trainees and plastic surgery departments and radiology departments due to protocol updates. Hospital administration will consider cost-effectiveness, while patients will experience changes in their care and recovery.

#### Guidelines/protocol

A radiologist and radiographer interviewed both highlighted the lack of code, guidance and protocol for IOUS and WGL, proving difficult to formally document the techniques used. The decision to request additional localisation after pre-operative imaging relies on the surgeon. IOUS techniques are then adapted to each patient’s case and depend on the techniques and collaborative efforts of the surgeon and radiologist. There is a SOP for WGL, but no SOP is available for IOUS use. A consultant radiologist or experienced fellow must complete the IOUS, given the technique adopted and the complex anatomy involved. This was evident, given one IOUS case could not be included as the registrar could not localise the lesion.

#### Localisation time

The radiologist mentioned the lack of certainty in procedure duration for the IOUS technique, averaging 30 min to complete, depending on the procedure, compared to WGL, which always takes around 15 min. Additionally, the radiologist described the additional coordination required across surgery and radiology for IOUS in confirming lesion excision.

#### Equipment

The USS required for WGL and IOUS localisation are readily available in hospitals. The Samsung prestige ultrasound used in WGL was reported to have a high resolution and a frequency range of 2.0–9.0 MHz. The Venue Go portable USS typically used intraoperatively, has a relatively higher resolution for superficial structures, a 39 mm field of view, a frequency range of 5.0–13.0 MHz and a penetration depth of 5 mm. Both USS are likely to provide greater accuracy in localising shallower lesions than pre-operative imaging. The radiographer mentioned the Venue Go may be more limited in visualising lesions in patients with higher body mass index patients or patients with deeper metastatic lesions given its higher frequency; however, its use intraoperatively means reimaging following excision is possible, allowing for further imaging of deeper structures and the smaller probe allows for greater manoeuvrability, particularly in challenging locations and deeper lesions. The radiographer noted that the sensitivity and specificity of machines are based on the context of use and cannot be directly compared. The accuracy of IOUS is dependent on the experience and skill set of the radiologist and surgeon, especially for complex anatomical locations.

#### Duration with radiologist

One radiologist approximated an average 15-minute duration with a radiologist for WGL compared with the hour-long duration blocked out for IOUS-guided lesion localisation.

## Discussion

Overall, this study highlighted the beneficial use of IOUS (Table 5). IOUS maintained patient comfort due to the minimal anxiety, discomfort and pain. Intraoperative reassurance on complete resection improved surgeon confidence, particularly near anatomically challenging locations. Margin and reoperation rates were higher in IOUS localisation and complication rates were lower for IOUS than WGL. This contrasts with a meta-analysis by Davet et al., proved lower margin positivity and reoperation rates in IOUS versus WGL [21] but it is important to acknowledge the limited follow-up period in our study. IOUS was linked to lower equipment and running costs, with the potential for further savings and easier implementation if surgeons were trained to perform IOUS imaging without radiologist assistance. Therefore, training courses for surgeons in IOUS would help to reduce reliance on radiology and cut costs. The USS probe used in WGL has greater penetration for deep lesions compared with the probe used for IOUS; however, the IOUS probe has a higher resolution for superficial structures and the limited imaging of deeper lesions can be overcome by reimaging deeper at the site following lesion excision [29, 30]. The applicability in using IOUS across various sites was demonstrated, unlike WGL, which had limited use, especially near important structures given concerns of wire-associated damage. For implementation, an SOP or general guidance around IOUS use is necessary; however, this study demonstrates that implementation of this technique is feasible and sustainable given the 19 cases presented. An SOP is required for implementation at scale to ensure consistency in use and standardisation, optimise patient safety and allow for multi-centre comparisons to refine the technique over time.

**Table 5:** Comparison of the benefits and drawbacks of Intraoperative ultrasound versus preoperative wire-guided localisation

Localisation method	Benefits	Drawbacks
Intraoperative ultrasound	- Non-invasive method - Cheaper set-up and running costs - Realtime guidance - Visualises anatomically challenging locations - Minimises healthy tissue resected. - Ability to rescan to rule out residual disease	- Requires experienced consultant radiologist - Longer duration with radiologist required - Extended duration of surgery - No standard operating procedure available
Preoperative wire-guided [31]	- Standard operating procedure in place - Shorter duration of surgery - Deep marking capability	- Invasive method which can cause pain, discomfort, bleeding and infection. - No ability to rescan intraoperatively - Wire dislodgment risk

### Strengths and limitations

This is the first quality improvement study in the UK study to compare IOUS versus WGL. Variations in locations of excisions demonstrated the applicability of IOUS use. One of the main limitations of this study is the limited sample size of both IOUS and WGL groups, reflecting its single-centre design. This may reduce statistical power and limit the strength and generalisability of the study’s conclusions. WGL cases performed in the last 3 years could only be captured since its on-site implementation was 3 years ago, unlike IOUS data, which could be captured over the last 8 years. Therefore, a direct comparison and conclusion cannot be made regarding the positive margin rates and reoperation rates, given the limited data, varied cancer types and sites and limited follow-up with WGL. Not all IOUS cases may have been captured, given the less reliable method of manually acquiring the data, given this technique’s novelty, and thus lack of IOUS code. To account for this limitation, robust methods of manually filtering electronic and diaries were used. Another limitation is that this study assumes complications and same-site re-excision are due to the localisation method alone; however, other factors such as tumour characteristics, staging, surgical technique and pathological assessment may play a role. A further study limitation is the lack of patient-reported outcomes for comfort regarding each procedure.

### Future work

Future work will address this study’s limitations with a larger cohort, longer follow-up and analysis across multiple centres. Further analysis is necessary to capture 10-year IOUS use versus 10-year WGL use to allow for more comparisons across more cases and identify whether WGL results in residual disease along the site of wire travel. Patient and public involvement was not included in the scope of this study, but is an important area of future research, as accounts regarding patient comfort and safety related to the procedure were acquired through surgeon reflections, given the retrospective study nature. A future prospective study including patient-reported outcomes and a survey study exploring patients’ preferences between the techniques and experiences, such as their comfort, is necessary. Following the provision of a standardised protocol, future work should compare IOUS use across hospital sites to establish the generalisability of findings.

## Conclusion

Overall, although IOUS use for localising oligometastatic disease extended the duration of surgery, it appeared to be less invasive, more accessible, potentially more cost-effective and easier to implement compared with WGL. IOUS was associated with generally low positive margin rates and re-excision rates. These findings suggest that IOUS may represent a promising alternative to WGL for lesion localisation, offering real-time intraoperative guidance. The development of SOPs and structured training may facilitate implementation and optimise the use of IOUS in clinical practice. A multi-site regional study with larger cohorts and longer follow-up is necessary to determine the generalisability of findings prior to national implementation in clinical practice.

## Data Availability

Data analysed during this study is included in the tables in this article.
